# *Rhaponticum acaule* (L) DC essential oil: chemical composition, *in vitro* antioxidant and enzyme inhibition properties

**DOI:** 10.1186/s12906-018-2145-5

**Published:** 2018-03-05

**Authors:** Habib Mosbah, Hassiba Chahdoura, Jannet Kammoun, Malek Besbes Hlila, Hanen Louati, Saoussen Hammami, Guido Flamini, Lotfi Achour, Boulbaba Selmi

**Affiliations:** 1 Laboratory of Bioresources: Integrative Biology and Valorization, Higher Institute of Biotechnology of Monastir, Avenue Taher Hadded BP 74, 5000 Monastir, Tunisia; 2 Laboratory of Biochemistry and Enzymatic Engineering of Lipases, ENIS, 3038 Sfax, Tunisia; 3 Laboratory of Transmissible Diseases and Biological Active Substances, Faculty of Pharmacy, Avicenne Avenue, 5000 Monastir, Tunisia; 4 Research Unit Applied Chemistry and Environment (UR13ES63), Faculty of Sciences of Monastir, Environment Avenue, 5019 Monastir, Tunisia; 5 Dipartimento di Farmacia, Via Bonanno 6, 56126 Pisa, Italy; 6 Centro Interdipartimentale di Ricerca “Nutraceutica e Alimentazione per la Salute”, Università di Pisa, Pisa, Italy

**Keywords:** α-glucosidase, Antioxidant activity, Chemical composition, Pancreatic lipase inhibition, *Rhaponticum acaule* essential oil, Xanthine oxidase

## Abstract

**Background:**

α-glucosidase is a therapeutic target for diabetes mellitus (DM) and α-glucosidase inhibitors play a vital role in the treatments for the disease. Furthermore, xanthine oxidase (XO) is a key enzyme that catalyzes hypoxanthine and xanthine to uric acid which at high levels can lead to hyperuricemia which is an important cause of gout. Pancreatic lipase (PL) secreted into the duodenum plays a key role in the digestion and absorption of fats. For its importance in lipid digestion, PL represents an attractive target for obesity prevention.

**Methods:**

The flowers essential oil of *Rhaponticum acaule* (L) DC (*R. acaule*) was characterized using gas chromatography-mass spectrometry (GC-MS). The antioxidant activities of *R. acaule* essential oil (RaEO) were also determined using 2,2’-azinobis-3-ethylbenzothiazoline-6-sulfonic acid (ABTS), reducing power, phosphomolybdenum, and DNA nicking assays. The inhibitory power of RaEO against α-glucosidase, xanthine oxidase and pancreatic lipase was evaluated. Enzyme kinetic studies using Michaelis-Menten and the derived Lineweaver-Burk (LB) plots were performed to understand the possible mechanism of inhibition exercised by the components of this essential oil.

**Results:**

The result revealed the presence of 26 compounds (97.4%). The main constituents include germacrene D (49.2%), methyl eugenol (8.3%), *(E)*-β-ionone (6.2%), β-caryophyllene (5.7%), *(E,E)*-α-farnesene (4.2%), bicyclogermacrene (4.1%) and *(Z)*-α-bisabolene (3.7%). The kinetic inhibition study showed that the essential oil demonstrated a strong α-glucosidase inhibiton and it was a mixed inhibitor. On the other hand, our results evidenced that this oil exhibited important xanthine oxidase inhibitory effect, behaving as a non-competitive inhibitor. The essential oil inhibited the turkey pancreatic lipase, with maximum inhibition of 80% achieved at 2 mg/mL. Furthermore, the inhibition of turkey pancreatic lipase by RaEO was an irreversible one.

**Conclusion:**

The results revealed that the RaEO is a new promising potential source of antioxidant compounds, endowed with good practical applications for human health.

## Background

Essential oil is a hydrophobic liquid extracted from various parts of plants such as flowers, leaves, stems and roots [1]. Due to its aromatic characteristic, essential oil has long been used in the food and cosmetic industries as a flavoring agent [2]. Furthermore, many essential oils exhibit antioxidant properties, which can have a positive effect on biological systems [3, 4] as well as on food production by preventing oxidation [5].

For a long time, essential oils have been the basis of traditional medicine in many countries [6, 7]. They are used with many biological properties including bactericidal, virucidal, fungicidal, antiparasitical, insecticidal, as well as with other medicinal properties such as analgesic, sedative, antiinflammatory, spasmolytic, and locally anesthetic remedies [8, 9]. At present, promising researches have been reportedly using essential oils in medicinal products for human health [10]. These recent works have shown the importance of essential oils in treating other diseases like respiratory tract, digestive system, gynecological, andrological, endocrine, cardiovascular, nervous system, and skin infections. Many of them have shown anticancer activities, too [11].

The *Rhaponticum* genus which belongs to the Asteraceae family is among the aromatic plants. This genus consists of about 30 species that are spread over the world [12]. As shown by the literature, several species of this genus are used in the traditional medicine. Indeed, the root of *Rhaponticum uniflorum* has been used for the treatment of fever and intoxication. It has been found that this species possesses anti-atherosclerotic activity and inhibits the lipids peroxidation [13]. On the other hand, *Rhaponticum carthamoides* has long been employed in cases of overstrain and common weakness after illness [14]. Actually, the extracts and isolated compounds from rhizomes and roots of this species are used for their adaptogenic and tonic properties in various dietary supplements or nutraceutical preparations to eliminate physical weakness, promote muscle growth, and treat impotency [15].

*Rhaponticum acaule* (L) DC, also known as *Leuzeaacaulis* L. or *Centaurea chamaerhaponticum Ball*., is one of the most remarkable aromatic plants having an earlier spring flowering, from January to March. It grows wild in rosette on the slopes and in sandy pastures. It is a North African endemic species, distributed in the north and central areas of Tunisia.

The only work published about the chemical composition of the aerial parts essential oils of *R. acaule* reported its richness in oxygenated sesquiterpenes (21.3%), aromatic compounds (23.6%) and diterpenoids (23.7%) [16]. In the same work, it was also found that the essential oil exhibited an interesting antibacterial activity [16]. However, to the best of our knowledge and according to literature, there are no reports on the antioxidant activities and enzyme inhibition effects of the essential oil from *R. acaule*.

Therefore, the goal of this study was to establish the chemical composition, the antioxidant effects of the essential oil of *R. acaule* collected in a very different habitat of Tunisia and its inhibitory power against α-glucosidase, xanthine oxidase and pancreatic lipase. Enzyme kinetic studies using Michaelis-Menten and the derived Lineweaver-Burk (LB) plots were performed to understand the possible mechanism of inhibition exercised by the components of this essential oil.

## Methods

### Chemicals reagents and enzymes

2,2’-azinobis-3-ethylbenzothiazoline-6-sulfonic acid (ABTS), trichloroacetic acid (TCA), potassium ferricyanide [K_3_Fe(CN)_6_], ferric chloride (FeCl_3_), Folin–Ciocalteu reagent (FC reagent), metallic magnesium, hydrochloric acid (HCl), sulphuric acid (H_2_SO_4_), acetic anhydride, sodium hydroxide (NaOH), aluminum chloride (AlCl_3_)_,_ *Aspergillus niger* α-glucosidase, Buttermilk grade I xanthine oxidase, *p*-nitrophenyl-α-D-glucopyranoside (pNPG), acarbose, *p*-nitrophenyl phosphate (pNPP) and xanthine were purchased from Sigma-Aldrich (St. Louis, MO, USA). All other chemicals used for the analyses were also obtained from Sigma-Aldrich. Turkey pancreatic lipase (TPL) was purified as described by Sayari and colleagues (Sayari et al. 2000). THL was a generous gift from Hoffmann la Roche (Basel, Switzerland.

### Plant material and essential oil extraction

The plants were collected at the flowering stage in January 2015 from the area of M’Saken (Sousse-Tunisia). The plant material was identified by Pr. Fethia Harzallah Skhiri (High Institute of Biotechnology of Monastir, Tunisia). A voucher specimen (N°.Ra15) has been deposited in the Herbarium of the Laboratory of Bioressources: Biologie Integrative and Valorization, High Institute of Biotechnology of Monastir, University of Monastir, Tunisia.

An amount of 100 g of *R. acaule* flowers freshly collected were cut in small pieces and submitted to hydrodistillation for 5–6 h with 500 ml of boiling distilled water using a Clevenger-type apparatus [17] in accordance with the European Pharmacopeia. The distilled essential oil was dried over anhydrous sodium sulfate, transferred to sealed dark vials and stored at 4 °C until use. The yield (0.018%) was calculated based on the fresh weight of the sample.

### Analysis of the essential oil

Analytical GC: essential oil composition was established by gas chromatograph: HP 5890-series II equipped with flame ionization detectors (FID), HP-5 (30 m × 0.25 mm ID, 0.52 μm film thickness) fused silica capillary column, carrier gas nitrogen (1.2 mL/min). The temperature oven was programmed from 50 °C (1 min) to 280 °C at 5 °C/min (1 min). Injector and detector temperatures were 250 °C and 280 °C, respectively. 0.1 μL of 1% hexane solution was injected. The identification of the different components was carried out when comparing their retention times with those of pure authentic samples and by mean of their linear retention indices (L.R.I) relative to the series of *n*-hydrocarbons.

Analytical GC–MS: GC/EIMS analyses were carried out with a Varian CP-3800 gas-chromatograph equipped with a HP-5 capillary column (30 m × 0.25 mm; coating thickness 0.25 μm) and a Varian Saturn 2000 ion trap mass detector. The analytical conditions were: injector and transfer line temperatures 220 and 240 °C respectively; oven temperature programmed from 60 °C to 240 °C at 3 °C/min; carrier gas helium at 1 mL/min; injection of 0.2 μL (10% hexane solution); split ratio 1:30. Identification of the compounds was based on comparison of the retention times with those of authentic samples, comparing their linear retention indices relative to the series of *n*-hydrocarbons, and on computer matching against commercial (NIST 2014 and ADAMS) and home-made library mass spectra built up from pure substances and components of known essential oils and MS literature data [18–21].

### Antioxidant activity

#### ABTS radical scavenging activity assay

The antiradical activity was performed by the ABTS^**.+**^ free radical decolorization assay as developed by Re et al. [22] with minor modifications. The 2,2-azino-bis-3-ethylbenzothiazoline-6-sulfonic acid (ABTS) was prepared as aqueous stock solution (7 mM). The ABTS radical cations (ABTS^**.**+^) were produced by the reaction of the ABTS stock solution with 2.45 mM of K_2_S_2_O_8_. The mixture of assay was incubated for 15 h in the dark at room temperature.

Furthermore, to obtain 0.7 ± 0.02 units at 734 nm as absorption, the solution was diluted with ethanol. Samples were separately dissolved in ethanol to yield the following concentrations: (0.312, 0.625, 1.25, 2.5, and 5 mg/mL). In order to measure the essential oil antioxidant activity, 10 μL were added to 990 μL of diluted ABTS^**·+**^at various concentrations. The absorption was read after 20 min of incubation. The antioxidant power of each sample was expressed as the inhibition percentage (I %) calculated according to the following formula:

$$ \mathrm{I}\%=\left(\left({\mathrm{A}}_{\mathrm{blank}}\hbox{--} {\mathrm{A}}_{\mathrm{sample}}\right)/{\mathrm{A}}_{\mathrm{blank}}\right)\times 100 $$

Where A_blank_ represents the absorbance of the control reaction (containing all reagents except the test compound), while A_sample_ represents the absorbance of the test sample.

The essential oil concentration providing 50% of radical scavenging activity (EC_50_) was calculated from the graph of radical scavenging activity percentage against essential oil concentration and Trolox (3.12–50 μM) was used as a standard. Test was carried out in triplicate.

#### Reducing power assay

The RaEO reducing power was evaluated using the method described by Oyaizu [23]. The essential oil concentrations ranged from 0.3 to 10 mg/mL. Aliquot of 1 mL essential oil dissolved in methanol was mixed with 1 mL of 200 mM sodium phosphate buffer (pH 6.6) and 1 mL (1%) of potassium ferricyanide [K_3_Fe(CN)_6_]. The obtained mixture was incubated at 50 °C for 20 min, and then acidified with 1 mL of trichloroacetic acid (10%). At the last step, 0.25 mL of FeCl_3_ (0.1%) were added to this solution. Distilled water was used as blank and for control. Using UV spectrophotometer, absorption of this mixture was measured at 700 nm. Increased absorbance of the mixture designates the sample ferric reducing power capability. The essential oil providing 0.5 of absorbance (EC_50_) was calculated using the absorbance graph of at 700 nm against essential oil concentration. Trolox was used as a positive control. The obtained values are presented as the means of triplicate assay.

#### Phosphomolybdenum assay

Essential oil samples (200 μL) were mixed with 2 mL of the phosphomolybdenum reagent (600 mM sulfuric acid, 28 mM sodium phosphate, 4 mM ammonium molybdate) [24]. Then, the mixture was incubated at 95 °C during 90 min and cooled to room temperature. Subsequently the absorbance was measured at 695 nm. In order to estimate the percentage of molybdenum reduced by tested essential oil, a standard curve was constructed using ascorbic acid. EC_50_ (mg/mL) corresponds to the effective concentration at which the total antioxidant activity (TAA) was 50% and was obtained by interpolation from linear regression analysis. As a positive control, the ascorbic acid was used. The values are presented as the means of triplicate assay.

#### DNA nicking assay

In this test, the DNA was treated according to the method of Lee et al. [25]. A volume of 5 μl of essential oil at the concentration of 2 mg/mL was added to 2 μl of pGEM®-Tplasmid DNA (0.5 μg/well). The mixtures were then kept for 10 min at room temperature followed by the addition of 10 μl of Fenton’s reagent (3 mM H2O2, 50 μM L-ascorbic acid and 80 μM FeCl3). The obtained mixtures were then incubated at 37 °C during 5 min. Finally, the DNA incubated with or without essential oil was then analyzed on 1% (*w*/*v*) agarose gel electrophoresis and visualized under ultraviolet light.

### α-glucosidase inhibition assay and kinetics study

A spectrophotometric α-glucosidase assay was performed as previously described by Tao et al. [26] with slight modifications as detailed by Rengasamy et al. [27]. The mixture of α-glucosidase reaction contained 2.5 mM *p*-nitrophenyl-α-D-glucopyranoside (pNPG), 250 μL of essential oil in DMSO and 0.3 U/mL of α-glucosidase in phosphate buffer, pH 6.9. Pure control having 100% enzyme activity was conducted by replacing the essential oil with DMSO. Blank for pure control having 0% enzyme activity was conducted with DMSO and by replacing the enzyme with buffer. In positive controls, acarbose, which is clinically used as an α-glucosidase inhibitor, replaced the essential oil. Absorbance of the released *p*-nitrophenol (pNP) was measured (405 nm) and was considered proportional to the activity of the enzyme. Each sample was carried out in triplicate. Inhibition percentage by essential oil and acarbose were calculated using the following equation:

$$ \mathrm{Inhibition}\ \mathrm{percentage}\ \left(\%\right)=\left(1-\left({\Delta \mathrm{OD}}_{\mathrm{sample}}/{\Delta \mathrm{OD}}_{\mathrm{control}}\right)\right)\times 100. $$

The IC_50_, which is the necessary concentration of the sample to inhibit 50% of the enzyme, was determined.

For the kinetics study, the reaction mixture was performed as described above, except that the substrate concentration increased from 0.625 to 10 mM, and in the presence of different concentrations of essential oil (7.5, 15 and 30 μg/mL). The reaction was started by the addition of enzyme, and monitored at 405 nm, at 5 min intervals during 30 min. The initial reaction rates were determined using calibration curves constructed by varying *p*-nitrophenol concentrations. The results were used to construct Lineweaver–Burk plots to determine the type of inhibition, Michaelis-Menten constant (K_m_) and maximum velocity (V_max_) values. The values of inhibition constant (*Ki*) were determined from the secondary plots constructed using slopes or y-intercepts of Lineweaver–Burk plots. (Ki) expresses the equilibrium constant for the binding of RaEO to α-glucosidase.

In general, there are four types of enzyme inhibition: competitive, non-competitive, uncompetitive and mixed. For mixed-type inhibition, the Lineweaver-Burk equation in a double reciprocal form can be expressed as follows:

1 $$ 1/v={\mathrm{K}}_{\mathrm{m}}/{\mathrm{V}}_{\mathrm{m}\mathrm{ax}}\ \left(1+\left[\mathrm{I}\right]/{\mathrm{K}}_{\mathrm{i}}\right)\ 1/\left[\mathrm{S}\right]+1/{\mathrm{V}}_{\mathrm{m}\mathrm{ax}}\ \left(1+\left[\mathrm{I}\right]/{\upalpha \mathrm{K}}_{\mathrm{i}}\right) $$

Secondary plots can be constructed from

2 $$ \mathrm{Slope}={\mathrm{K}}_{\mathrm{m}}/{\mathrm{V}}_{\mathrm{m}\mathrm{ax}}+{\mathrm{K}}_{\mathrm{m}}\ \left[\mathrm{I}\right]/{\mathrm{V}}_{\mathrm{m}\mathrm{ax}}{\mathrm{K}}_{\mathrm{i}} $$

or

3 $$ \mathrm{Y}-\mathrm{intercept}=1/{{\mathrm{V}}_{\mathrm{max}}}^{\mathrm{app}}=1/{\mathrm{V}}_{\mathrm{max}}+1/{\upalpha \mathrm{K}}_{\mathrm{i}}{\mathrm{V}}_{\mathrm{max}}\ \left[\mathrm{I}\right] $$

Where *v* is the enzyme reaction rate in the absence and presence of essential oil. [I] and [S] are the concentrations of the inhibitor and substrate, respectively, α is the apparent coefficient.

### Xanthine oxidase inhibition assay and kinetics study

Xanthine oxidase activity was measured spectrophotometrically at 295 nm by continuously measuring uric acid formation, according to the protocol of Kong et al. [28]. The reaction mixture is composed of 250 μL of test solution, 300 μL of 70 mM phosphate buffer (pH 7.5) and 300 μL of substrate solution (150 μM xanthine in the same buffer). After preincubation at 25 °C during 15 min, the reaction was triggered by the addition of 150 μL enzyme solution (0.1 units/mL in 70 mM phosphate buffer (pH 7.5) freshly prepared before use. Pure control having 100% enzyme activity was conducted by replacing the essential oil with DMSO. Blank for pure control having 0% enzyme activity was conducted with DMSO and by replacing the enzyme with buffer. Allopurinol was used as a positive control. The RaEO was tested for xanthine oxidase inhibitory activity at various concentrations. Each sample was carried out in triplicate. The inhibitory activity was determined by IC_50_, which was obtained from percent inhibition calculated by the following equation:

Percent inhibition (%) = (1 − (ΔOD_sample_/ΔOD_control_)) × 100.

The IC_50_, which is the concentration of the sample required to inhibit 50% of the enzyme was determined for each sample.

For the kinetics study, the reaction mixture was as described above, except that the substrate concentration increased from 37.5 to 300 μM, and in the presence of different concentrations of RaEO (2.5, 3.75 and 5 μg/mL). The reaction was initiated by the addition of enzyme, and monitored at 295 nm and at 5 min intervals during 30 min. The obtained results were used to construct Lineweaver–Burk plots to determine the inhibition mode, Michaelis-Menten constant (K_m_) and maximum velocity (V_max_) values. The inhibition constant (Ki) value was calculated from the secondary plot constructed using Y-intercepts of Lineweaver–Burk plots. (Ki) expresses the equilibrium constant for the binding of RaEO to xanthine oxidase.

For a general analysis of non-competitive inhibition, the Lineweaver-Burk equation can be written in double-reciprocal form:

4 $$ 1/v={\mathrm{K}}_{\mathrm{m}}/{\mathrm{V}}_{\mathrm{m}\mathrm{ax}}\ \left(1+\left[\mathrm{I}\right]/{\mathrm{K}}_{\mathrm{i}}\right)\ 1/\left[\mathrm{S}\right]+1/{\mathrm{V}}_{\mathrm{m}\mathrm{ax}}\ \left(1+\left[\mathrm{I}\right]/{\mathrm{K}}_{\mathrm{i}}\right) $$

Secondary plots can be constructed from

5 $$ \mathrm{Y}-\mathrm{intercept}=1/{{\mathrm{V}}_{\mathrm{max}}}^{\mathrm{app}}=1/{\mathrm{V}}_{\mathrm{max}}+1/{\mathrm{K}}_{\mathrm{i}}{\mathrm{V}}_{\mathrm{max}}\ \left[\mathrm{I}\right] $$

The secondary replot of Y-intercept vs. [I] is linearly fitted assuming a single inhibition site or a sing class of inhibition sites.

### In vitro pancreatic lipase inhibition assay

#### Measurement of pancreatic lipase activity

The pancreatic lipase activity was measured titrimetrically with a pH-Stat (Metrohm, Switzerland) at pH 8.5 and 37 °C using olive oil emulsion as substrate and in the presence of 4 mM NaDC and Turkey pancreatic colipase previously purified according to Rathelot and colleagues [29]. One lipase unit corresponds to 1 μmol of fatty acid released per minute.

#### Pancreatic lipase inhibition test

In this assay, RaEO was dissolved in DMSO and was used to evaluate its inhibitory effect on lipase. To evaluate the pancreatic lipase inhibitory activity, TPL was preincubated at room temperature for 1 h with various RaEO concentrations. The reaction medium contained 20 μL of RaEO and 60 μL of enzyme. After preincubation, 40 μL from the reaction mixture were used to evaluate the residual pancreatic lipase activity, as previously indicated. Pure control having 100% enzyme activity was conducted by replacing the essential oil with DMSO. Blank for pure control having 0% enzyme activity was conducted with DMSO and by replacing the enzyme with buffer. On the other hand, THL was used as a positive control for this test. The lipase inhibition (% inhibition) was calculated in comparison with the initial activity, measured in the absence of inhibitors. The IC_50_ values were calculated from plots of log concentration of essential oil versus percentage inhibition curves using Sigma Plot 12.1 (IL, USA).

### Statistical analysis

The means and standard deviation (SD) of data were calculated from independent experiments. The IC_50_ (α-glucosidase, xanthine oxidase and pancreatic lipase inhibition) and EC_50_ (ABTS, reducing power and phosphomolybdenum methods) values were calculated by linear regression analysis and the limits of their confidence intervals were carried out under the normality assumption. Data analysis was carried out using an unpaired Student’s *t*-test. GraphPad Software (USA) was used to fit sigmoid curves models and data were analyzed using a statistical analysis computer software (Graphpad Instat v.3.0a for MacIntosh, San Diego, CA, USA). For all statistical tests, *P*-values that were less than 0.05 were considered to be significant.

## Results

### Chemical composition of the *R. acaule* essential oil

The total yield of the volatile fraction obtained from aerial part of *R. acaule* was 0.018% (*w*/w). The obtained essential oil was yellow with a pleasant odour. The chemical composition of the essential oil obtained from the aerial part was investigated using both GC and GC/MS techniques. The percentages and the retention indices of the identified oil components were listed in Table 1 in the order of their elution on the HP-5MS column. As shown in this table, 26 components were identified which represent 97.4% of the total oil. The major constituents were germacrene D (49.2 ± 1.1%), methyl eugenol (8.3 ± 0.28%), *(E)*-β-ionone (6.2 ± 0.18%), β-caryophyllene (5.7 ± 0.17%), *(E,E)*-α-farnesene (4.2 ± 0.1%), bicyclogermacrene (4.1 ± 0.12%) and *(Z)*-α-bisabolene (3.7 ± 0.08%). Table 1 showed also that the essential oil was characterized by the dominance of sesquiterpene hydrocarbons (74.2%).

**Table 1:** Composition of the essential oil of *R. acaule*

Constituents^a^	l.R.I.^b^	(%)^c^
*(E,E)*-2,4-heptadienal	1012	0.2 ± 0.002
Linalool	1101	0.4 ± 0.010
Nonanal	1104	0.2 ± 0.004
Methyl chavicol (synonim estragole)	1197	0.5 ± 0.010
Decanal	1206	0.6 ± 0.014
1-tridecene	1292	0.8 ± 0.010
Eugenol	1358	1.8 ± 0.014
α-copaene	1377	1.3 ± 0.011
β-cubebene	1391	0.6 ± 0.010
β-elemene	1392	1.8 ± 0.018
Methyl eugenol^d^	**1403**	**8.3 ± 0.280**
β-caryophyllene	**1419**	**5.7 ± 0.170**
β-copaene	1430	0.2 ± 0.003
*cis*-α-ambrinol	1437	0.9 ± 0.017
α-humulene	1455	1.7 ± 0.016
2-methyltetradecane	1462	0.8 ± 0.012
γ-curcumene	1481	0.5 ± 0.010
Germacrene D	**1482**	**49.2 ± 1.100**
*(E)*-β-ionone	**1487**	**6.2 ± 0.180**
Bicyclogermacrene	**1496**	**4.1 ± 0.120**
α-muurolene	1499	0.4 ± 0.011
*(Z)*-α-bisabolene	**1506**	**3.7 ± 0.080**
*(E,E)*-α-farnesene	**1508**	**4.2 ± 0.100**
δ-cadinene	1524	0.8 ± 0.014
Elemicin	1555	0.8 ± 0.015
Dendrolasin	1580	1.5 ± 0.017
Oxygenated monoterpenes		0.4 ± 0.010
Sesquiterpene hydrocarbons		74.2 ± 1.663
Oxygenated sesquiterpenes		1.5 ± 0.017
Phenylpropanoids		11.4 ± 0.319
Apocarotenes		7.1 ± 0.197
Non-terpene derivatives		2.8 ± 0.042
Total identified		97.4 ± 2.248

^a^Identification of compounds was made by the calculation of their L.R.I and by GC–MS analysis

^b^LRI: linear retention indices (HP-5 column)

^c^%: Percentage calculated by GC-FID on non-polar capillary column HP-5

^d^Main compounds in bold

### Antioxidant activity

#### ABTS radical scavenging activity

The ABTS-radical-scavenging activity test measures the capacity of the sample to decrease the amount of ABTS^•+^ cation radical in the solution. This method is widely used to evaluate the antioxidant activity of several substances since it can be applied both to liposoluble and hydrosoluble substances. This can be explained by the fact that this method evaluates the scavenging potential of lipid or hydrogen peroxyl radicals in an aqueous phase [30]. In our case, it was observed that the *R. acaule* essential oil has a dose-dependent ABTS-radical-scavenging action, with an EC_50_ of 0.929 ± 0.118 mg/mL (Table 2).

**Table 2:** Antioxidant activity of the *R*. *acaule* essential oil

Assay	EC_50_ values (mg/mL)		
	RaEO	Trolox	Ascorbic acid
^+^ABTS	0.929 ± 0.118	0.037 ± 0.0002^a^	–
^++^FRAP	0.604 ± 0.021	0.018 ± 0.0001^a^	–
^+++^TAA	0.167 ± 0.019	–	0.068 ± 0.0001^a^

^+^EC_50_ (mg/mL): effective concentration at which 50% of ABTS radicals are scavenged

^++^EC_50_ (mg/mL): effective concentration at which the absorbance is 0.5

^+++^EC_50_ (mg/mL): effective concentration at which the total antioxidant activity (TAA) was 50%

Effect of Trolox or Ascorbic acid used as standards, were measured in the same conditions than the RaEO. All experiments were performed in triplicate and the results were expressed as the mean ± standard deviation (SD). Statistical comparisons were performed through an unpaired Student’s *t*-test using GraphPad InStat version 3.0a for MacIntosh

^a^: *p* < 0.05 vs RaEO

#### Reducing power

The reducing capacity of products is an important indicator of their antioxidant effect because it evaluates the ability of the sample to donate hydrogen atoms and to interfere with the free-radical chain reaction [31]. A high absorbance indicates a higher antioxidative activity. In this work, the ability of the RaEO to reduce Fe^3+^ to Fe^2+^ was conducted. Our results show that the reducing capacity increased with the essential oil concentration in a dose-dependent manner with an EC_50_ value of 0.604 ± 0.021 mg/mL (Table 2).

#### Phosphomolybdenum assay

In this assay, the antioxidant capacity of RaEO was measured using the phosphomolybdenum method as described by Prieto et al. [24]. The basic principle to assess the antioxidant capacity through this assay includes the reduction of Mo (VI) to Mo (V) by the antioxidant compounds contained in the oil. Similarly to the two methods above, this test further confirms that the essential oil was very effective in the reduction of Mo (VI) to Mo (V) (EC_50_ = 0.167 ± 0.019 mg/mL).

#### DNA nicking assay

**Fig. 1:**
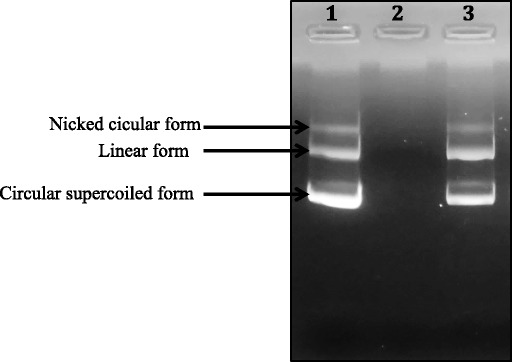
Gel electrophoresis pattern of the plasmid pGEM®-T incubated with Fenton’s reagent in the presence or absence of RaEO. Lane 1: Untreated control: native pGEM®-T DNA (0.5 μg); Lane 2: DNA sample incubated with Fenton’s reagent; lane 3: Fenton’s reagent + DNA + 2 mg/mL of RaEO

Hydroxyl radicals are known for their capacity to cause oxidative damage to biomolecules such as lipids, proteins and DNA. The antioxidant effect of RaEO using DNA nicking assay is presented in Fig. 1. The untreated plasmid (lane 1) represents three conformations: the nicked, the linear and the supercoiled forms. On the other hand, incubation of plasmid DNA with Fenton’s reagent resulted in the total degradation of the plasmid and the loss of the three bands (lane 2). We can notice essentially that the pre-incubation of DNA with the essential oil at 2 mg/mL, before the incubation with Fenton’s reagent, protected the plasmid DNA against oxidation. Furthermore, the RaEO, with 2 mg/mL as concentration shows significant protective effect with important conservation of the supercoiled DNA band intensity.

### α-glucosidase inhibition assay and kinetics study

According to literature, few reports exist for the inhibition activity of α-glucosidase by essential oils. The low IC_50_ values obtained in this assay indicated a high inhibition activity. From our results, RaEO shows interesting antidiabetic activity with IC_50_ value of 6.7 ± 0.10 μg/mL which is 42-fold lower than that of the commercial inhibitor acarbose (280 ± 10.01 μg/mL).

**Fig. 2:**
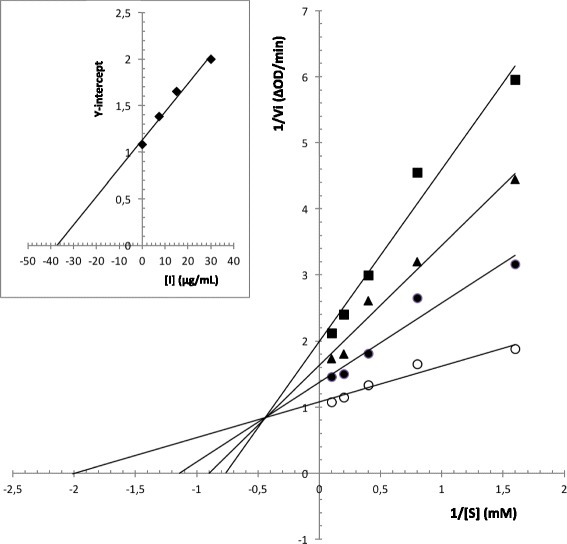
Lineweaver-Burk plot of α-glucosidase inhibition at different substrate [S] concentrations in absence (○) or presence of various concentrations (●: 7.5 μg/mL; ▲: 15 μg/mL and ■: 30 μg/mL) of RaEO. Insert: Secondary replot of Y-intercept against inhibitor concentrations to calculate the inhibition constant (Ki). Each point is the average value from three independent experiments

To investigate the type of enzyme inhibition and to determine the inhibition constant (Ki), the kinetics was performed according to the procedure detailed in the Materials and Methods section. The Lineweaver–Burk plots analysis indicated that the lines coincided on a point on the left side of the *Y*-axis and intercepted both the *Y*- and the *X*-axes at different points. What can be also observed are a decrease in apparent V_max_ value and an increase in the value of apparent K_m_, indicating that the nature of inhibition is of mixed-type (Fig.2).

Moreover, from Fig. 2, it can be noted that the intersecting lines on the graph converge at the left side of the Y-axis and above the X-axis, indicating that the value of α (a constant that defines the degree to which inhibitor binding affects the affinity of the enzyme for substrate) is greater than 1 [32]. This confirms that the inhibitor prefers binding to the free enzyme rather than to the enzyme substrate complex. Therefore, the mode of inhibition caused by the essential oil is a mixed-type one, but it seems that it has strong competitive components. The inhibition constant (Ki) value derived from the secondary plot (Fig. 2) was 37.5 μg/mL.

### Xanthine oxidase inhibition assay and kinetics study

The RaEO was tested for xanthine oxidase inhibitory activity at various concentrations and thereafter the IC_50_ value was deduced. It can be observed that this essential oil has a very interesting IC_50_ value (2.20 ± 0.10 μg/mL), which is comparable to that of the reference compound, allopurinol (2.6 ± 0.16 μg/mL).

**Fig. 3:**
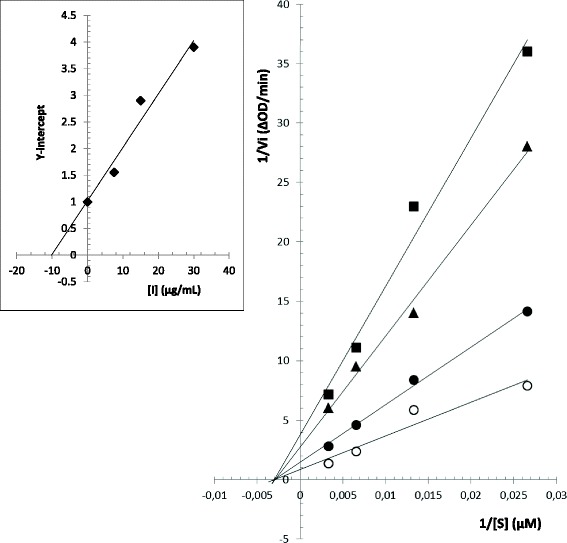
Lineweaver-Burk plot of xanthine oxidase inhibition at different substrate [S] concentrations in absence (○) or presence of various concentrations (●: 2.5 μg/mL; ▲: 3.75 μg/mL and ■: 5 μg/mL) of RaEO. Insert: Secondary replot of Y-intercept against inhibitor concentrations to calculate the inhibition constant (Ki). Each point is the average value from three independent experiments

Under non-inhibitory conditions, kinetic parameters for xanthine oxidase were K_m_ = 0.003 μM and V_max_ = 1.052 ΔOD/min. Assays with the essential oil induced a modification on V_max_ (from 0.26 to 0.66 ΔOD/min), whereas the K_m_ value was not changed (Fig. 3). As illustrated in Fig. 3, the slope and the vertical axis intercept increase with increasing essential oil concentration (2.5, 3.75 and 5 μg/mL). This result indicates that the essential oil components affected the velocity of the reaction catalyzed by xanthine oxidase, without affecting the Michaelis constant (K_m_). From these results, we can conclude that this inhibition is a non-competitive one, indicating that the components of the essential oil bind to a site other than the active one of the enzyme, without competing with the substrate.

The Ki value derived from the secondary plot (Fig. 3) was 10 μg/mL, indicating that this essential oil tended to bind more easily to the xanthine oxidase. Indeed, smaller value of inhibition constant indicates stronger inhibition, which explains that the inhibitor-enzyme binding affinity exceeds the binding affinity of the enzyme-substrate.

### Anti-lipase activity of *R. acaule* essential oil

In this work, TPL was used to measure the anti-lipase activity of RaEO. This enzyme was totally purified and its relation structure–function is previously determined [33]. These authors showed that the biochemical properties and structures of TPL are similar to those of mammals. For this reason, TPL was used instead of the mammalian one. The anti-lipase activity of the essential oil was conducted using two methods, which differ in the order of the inhibitor addition.

#### Method a: Preincubation of lipase with inhibitor

**Fig. 4:**
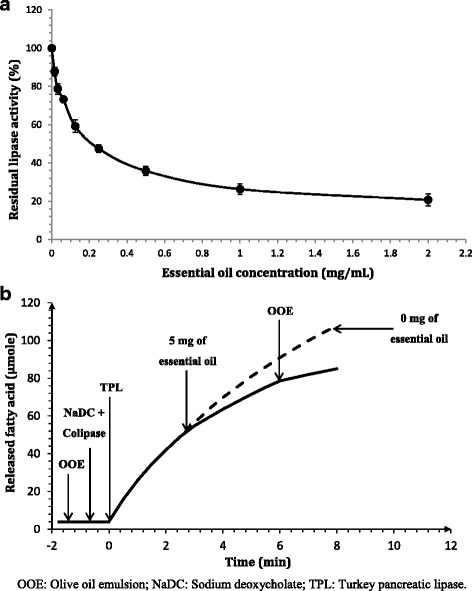
**a**: Residual pancreatic lipase activity at various concentrations of RaEO. Lipase activity was measured using olive oil as substrate, in the presence of 4 mM of NaDC. Results are expressed as means ± S.D., *n* = 3. **b**: Kinetic experiment of hydrolysis of olive oil emulsion by TPL. The kinetic curve was recorded continuously during the automated titration of liberated fatty acids under standard conditions using pH-stat apparatus. The arrows indicate the successive injections into the reaction medium of olive oil emulsion (OOE), (NaDC + colipase), TPL and RaEO

The aim of this method was to test, in an aqueous medium and in the absence of the substrate, the possible reactions between the pancreatic lipase and the inhibitor. The residual lipase activity was investigated at different concentrations of essential oil (0.05 to 2.00 mg/mL) (Fig. 4a). As shown by the figure, a dose–response curve was obtained, with maximum inhibition (80%) achieved at 2.00 mg/mL of essential oil. The inhibitory activity of the essential oil expressed as IC_50_ value was about 0.22 ± 0.002 mg/mL, which is lower than that of pure THL (0.16 ± 0.001) used as a standard inhibitor against pancreatic lipase.

#### Method B: Inhibition during lipolysis

This method was performed to observe the behavior of the lipase (inactivation or not) at the lipid/water interface during the hydrolysis of the water-insoluble substrate. The hydrolysis rate of olive oil emulsion was measured at pH 8.5 with TPL in the presence of colipase and NaDC. The essential oil was then added during substrate hydrolysis and the residual activity was measured. As depicted by Fig. 4b, the rate of olive oil hydrolysis by TPL decreased rapidly. The addition of an excess of substrate (olive oil emulsion) at the end of the kinetic does not succeed in restoring the initial velocity of the enzyme, showing that the lipase inhibition was irreversible.

## Discussions

### Chemical composition of the *R. acaule* essential oil

According to the literature, the comparison of the chemical composition of our essential oil (RaEO) with that of *Rhaponticum carthamoides* roots studied by Skała et al. [34], revealed important similarity. In fact, these authors reported sesquiterpene hydrocarbons as the main class of chemicals in this essential oil (54.7%). Furthermore, Havlik et al. [35] noted that sesquiterpene lactones exist in all parts of *Rhaponticum carthamoides* while the sesquiterpene hydrocarbons were identified only in essential oil. On the other hand, Benyelles et al. [36] reported that the essential oil extracted from the roots of *R. acaule* was characterized by a large amount of alcohol derivatives (69.2%). Finally it is worth to note that for the essential oil of the aerial part of the same species (*R. acaule*), Boussaada et al. [16] described a composition richer in diterpenoids (23.7%), aromatic compounds (23.6%) and oxygenated sesquiterpenes (21.3%). However, these authors collected the plant material in the completely different habitat of Tunisia. Indeed, the differences between the chemical compositions of the essential oil obtained from the same species may be attributable to environmental factors (geographical, climatic, and seasonal), development stage and genetic variability [37–40]. Additionally, chemotypes and individual variability may appear within the same plant species, resulting in the differences in chemical compositions of the raw materials [41].

### Antioxidant activity

Despite the many publications on the chemical composition and antimicrobial activity of essential oils for the *Rhaponticum* genus, little information is available about their antioxidant activity [42]. Therefore, this is the first report about the antioxidant activity of the essential oil of *R. acaule*. Overall, according to the three tests used in this study (ABTS radical scavenging activity, Reducing power and Phosphomolybdenum assay), the obtained antioxidant activity of the RaEO might be attributed to the presence of high percentages of mainly germacrene D (49.2 ± 1.1%), methyl eugenol (8.3 ± 0.28%), (E)-β-ionone (6.2 ± 0.18%) and β-caryophyllene (5.7 ± 0.17%) (Table 1). Similarly to our results, some works reported that several essential oils having low phenolic compounds amounts also exhibits interesting antioxidant capacity [43, 44]. However, it’s difficult to correlate the antioxidant activity of a total essential oil to one or few active compounds. Both minor and major compounds should make a significant contribution to the activity of the oil.

### α-glucosidase inhibition

Among the available approaches, inhibition of α-glucosidase has appeared to be a potential therapeutic target for the treatment and prevention of type 2 diabetes mellitus (DM) [45]. For a long time plant food rich in polyphenols have been reported to cause effects similar to insulin in the utilization of glucose and act as good inhibitors of key enzymes like α-glucosidase associated with type 2 diabetes and lipid peroxidation in tissues [46]. On the other hand, according to literature, few reports exist for the inhibition activity of α-glucosidase by essential oils. In this study, our results revealed that the RaEO exhibited good antidiabetic activity which is 42-fold higher than that of acarbose used as commercial inhibitor. This interesting antidiabetic activity may be due to the large amount of sesquiterpene hydrocarbons, especially germacrene D, as hypothesized by Sarikurkcu et al. [47] using the essential oils of three *Phlomis* species.

### Xanthine oxidase inhibition

It is well known that allopurinol, an inhibitor of xanthine oxidase which is structurally related to xanthine, binds to the active site of this enzyme, causing its inhibition. This drug has been clinically used for more than 40 years. Unfortunately, the appearance of several side effects including fever, allergic reactions, skin rashes, hepatitis, and nephropathy limit the clinical use of allopurinol. For this reason, the search for novel natural xanthine oxidase inhibitors would be beneficial to treat gout and other diseases.

In our case, the significant xanthine oxidase inhibition exercised by the RaEO may be explained by the high amount of sesquiterpene hydrocarbons contained in this essential oil. Our results are in good agreement with those obtained by Kaurinovic et al. [43] with the essential oil of *Marrubium peregrinum* L., which is rich in β-caryophyllene, bicyclogermacrene and germacrene D.

### Pancreatic lipase inhibition

According to the literature, Asteraceae species are well documented for their antimicrobial activities, but to the best of our knowledge there are no studies about their pancreatic lipase inhibition ability. Thus, this is the first study conducted on the essential oil of *R. acaule* to inhibit pancreatic lipase. In this work, Turkey pancreatic lipase (TPL) was used to measure the anti-lipase activity of RaEO.

Using the method A (preincubation of lipase with inhibitor), the RaEO exhibited promising anti-lipase activity which is comparable with that of pure THL used as a standard inhibitor against pancreatic lipase. In the same way and according to the literature, the inhibition of pancreatic lipase by the essential oil of *Salvia judaica* (Lamiaceae) flowers was determined colorimetrically and compared with Orlistat used as a positive control by Afifi et al. [48]. The IC_50_ value (0.108 mg/mL) obtained in this study was very close to that obtained with RaEO (0.2 mg/mL). In this work, similarly to our essential oil, the aroma profile of the *Salvia judaica* (Lamiaceae) flowers was found to be mainly composed by sesquiterpene hydrocarbons (71.8%).

To determine the type of inhibition exerted by the RaEO on the TPL, the method B (inhibition during lipolysis) was used. Our results revealed that the obtained lipase inhibition was irreversible. Similar results have been reported by Gargouri et al. [49] using pure THL as inhibitor for porcine pancreatic lipase. In this case, the lipase hydrolysis rate of olive oil decreased rapidly to reach 10%, with final THL concentration of 200 μM. Gargouri et al. [50] have described two types of inhibition: the reversible inactivation obtained with amphiphiles compounds, such as detergents and some proteins. This inhibition is due to the modification of the interface quality, which prevents the binding of lipase to the interface [51]; the irreversible inhibition was obtained in the presence of colipase and bile salt and cannot be suppressed by renewal or excess of interface. The irreversible inhibition was obtained with specific reagents, such as THL, C12:0-TNB and E600 [49].

## Conclusion

In this work we studied a medicinal and aromatic plant of the Tunisian flora, in order to find new bioactive natural compounds. In this work we described the chemical composition of the essential oil from aerial part of *R. acaule*. The major compounds identified in this oil were germacrene D, methyl eugenol, *(E)*-β-ionone, β-caryophyllene, *(E,E)*-α-farnesene, bicyclogermacrene and *(Z)*-α-bisabolene. Our results also evidenced for the first time that this essential oil has interesting antioxidant and free radical-scavenging activities. Furthermore, in this work we have investigated for the first time the in vitro α-glucosidase inhibitory activities of RaEO with enzyme kinetics analyses. The results showed that the essential oil exhibited a strong inhibition on the α-glucosidase activity that is higher than acarbose, and the inhibition was a mixed-type mechanism. Furthermore, the inhibitory effect of this essential oil on xanthine oxidase was conducted. Lineweaver-Burk representation of the inhibition kinetics data proved that it was a non-competitive inhibitor of xanthine oxidase and its Ki value was 10 μg/mL. Finally, RaEO exhibited a high inhibitory effect against pancreatic lipase. This suggests that it is a promising essential oil for inactivating digestive lipase in order to decrease incidence of common diseases caused by diets rich in carbohydrates and fats.

However, further studies are needed to understand the origin of the activity. Particularly, major constituents of the essential oil need to be tested for their antioxidant, anti-α-glucosidase, anti-xanthine oxidase and anti-lipase activities. Furthermore, it is still worthwhile to investigate the other parts of *Rhaponticum acaule* as a natural source for essential oil composition or phytochemical studies.

## Abbreviations

ABTS: 2,2’-azinobis-3-ethylbenzothiazoline-6-sulfonic acid
OOE: Olive oil emulsion
PNPG: *P*-nitrophenyl-α-D-glucopyranoside
*R. acaule*: *Rhaponticum acaule* (L) DC
RaEO: *Rhaponticum acaule* essential oil
TAA: Total antioxidant activity
THL: Tetrahydrolipstatine
TPL: Turkey pancreatic lipase
